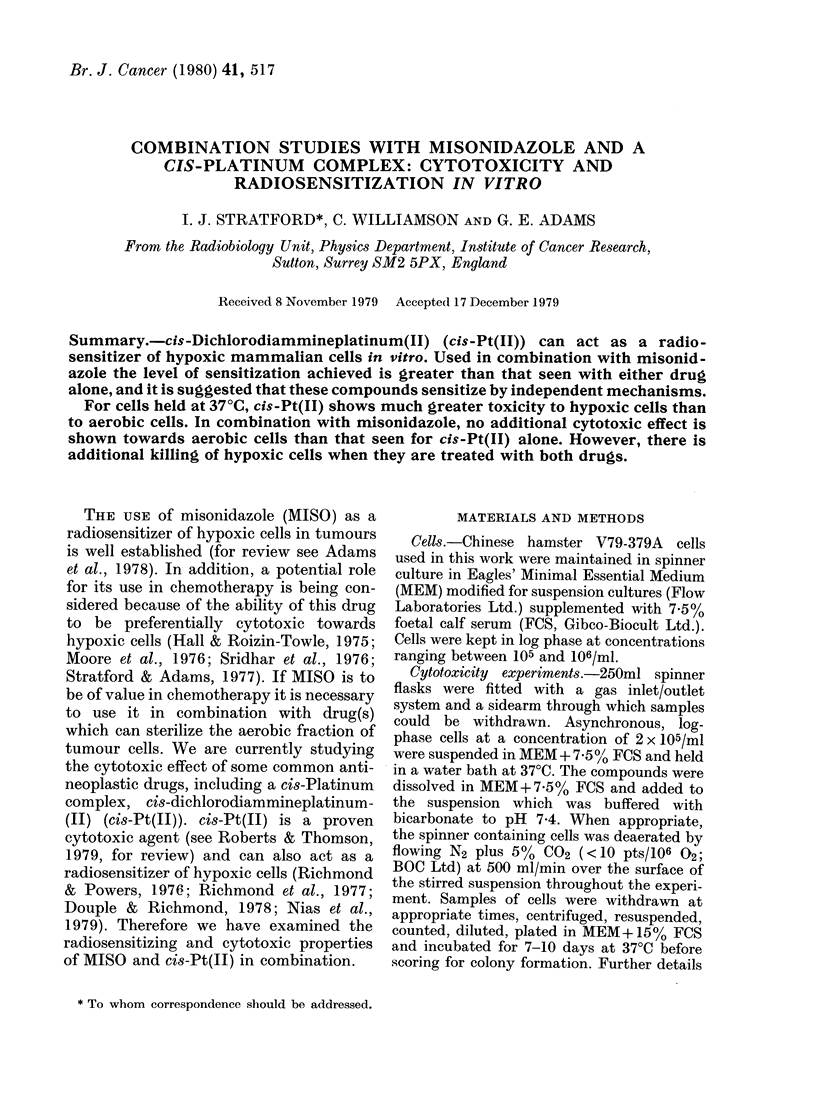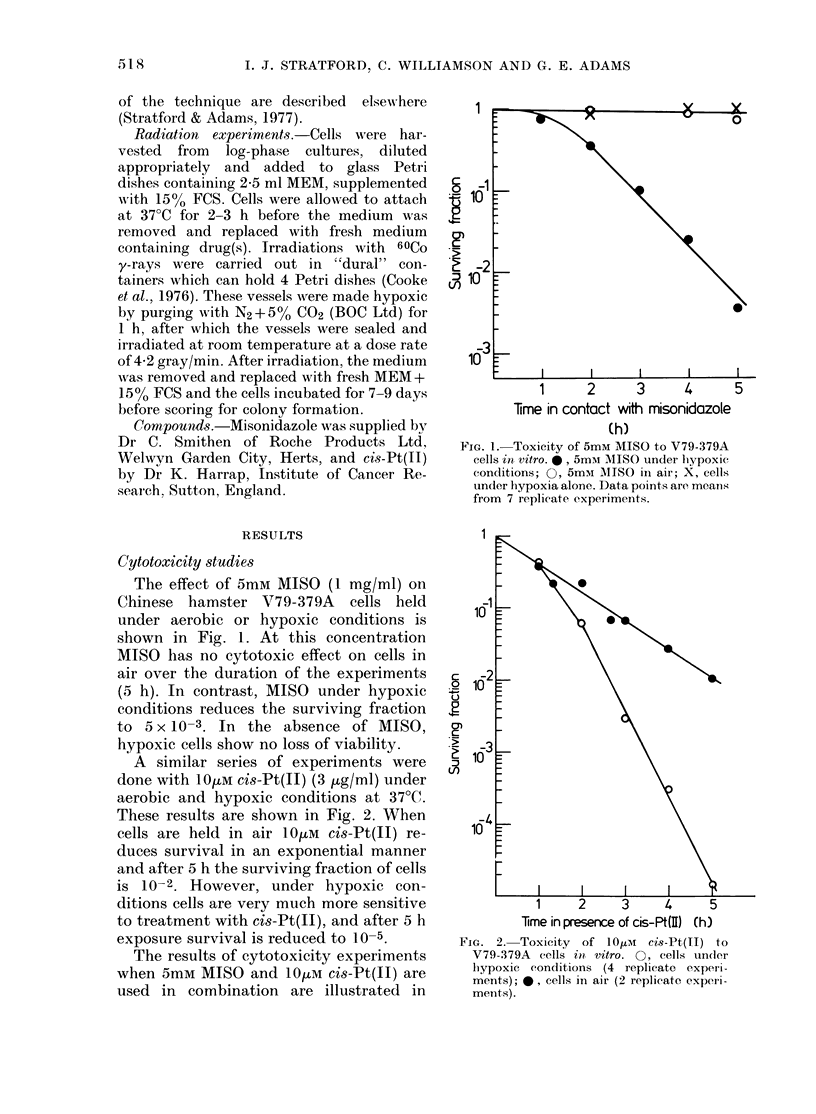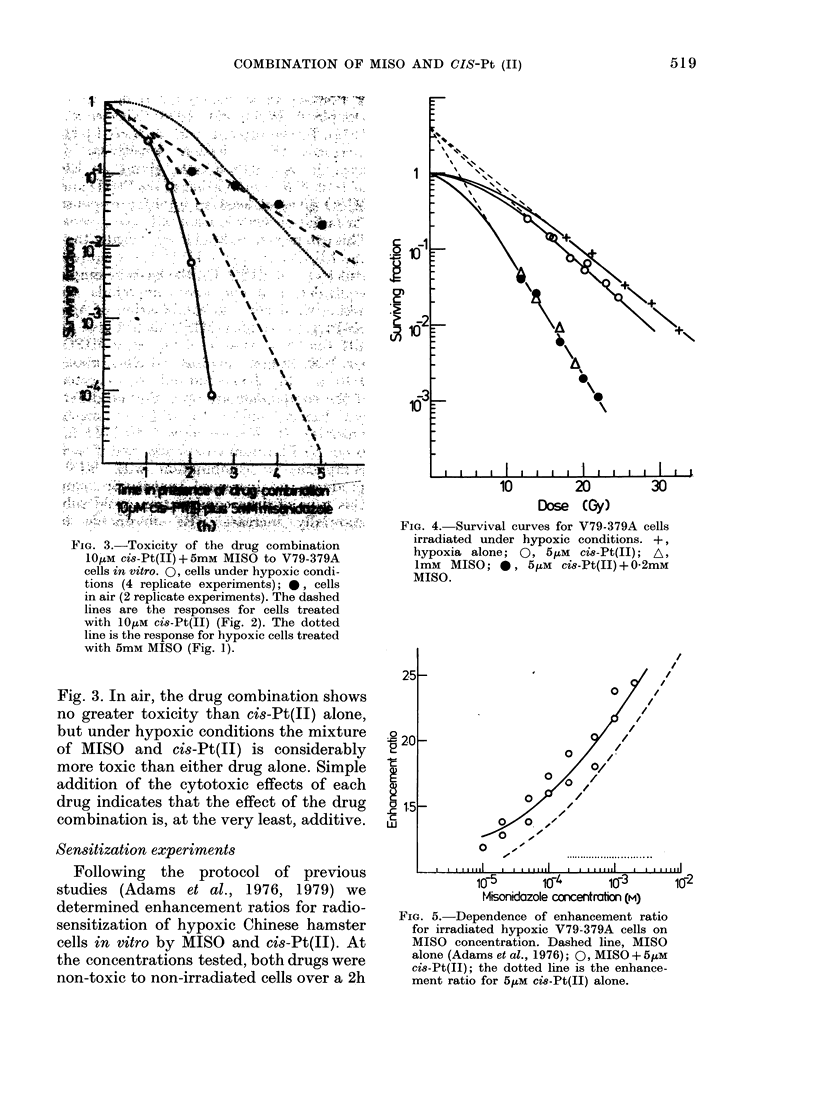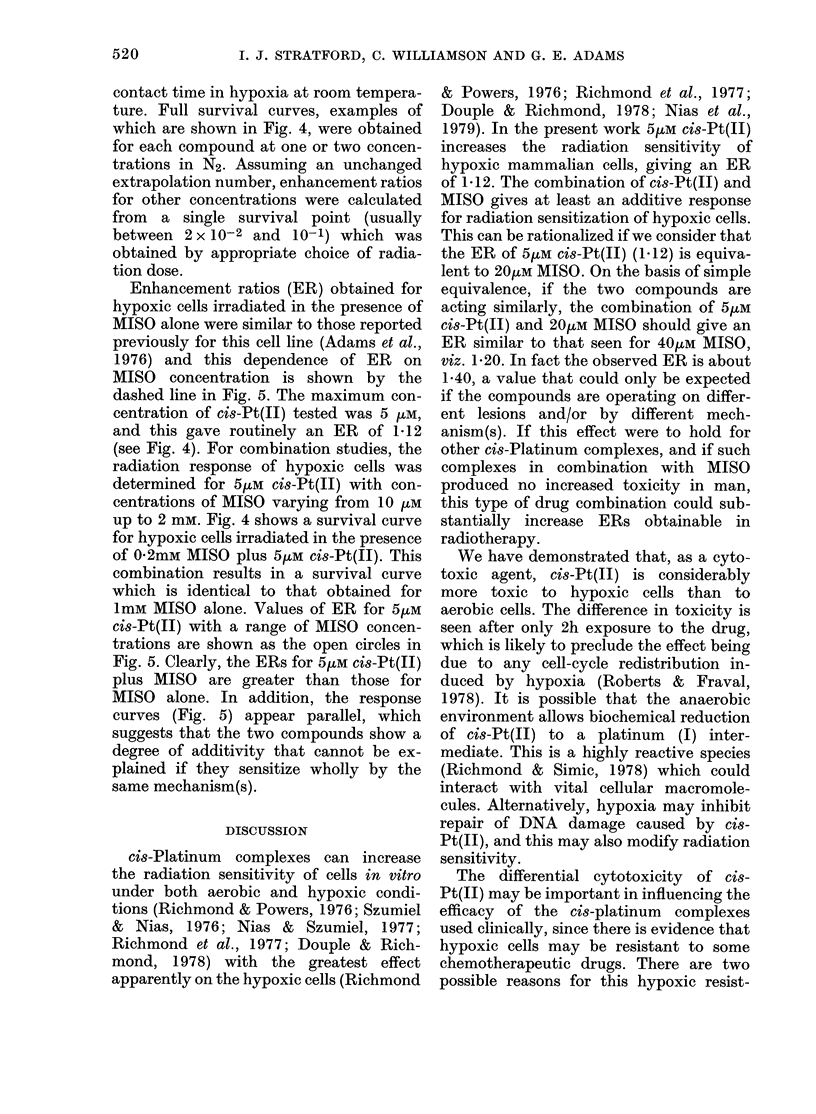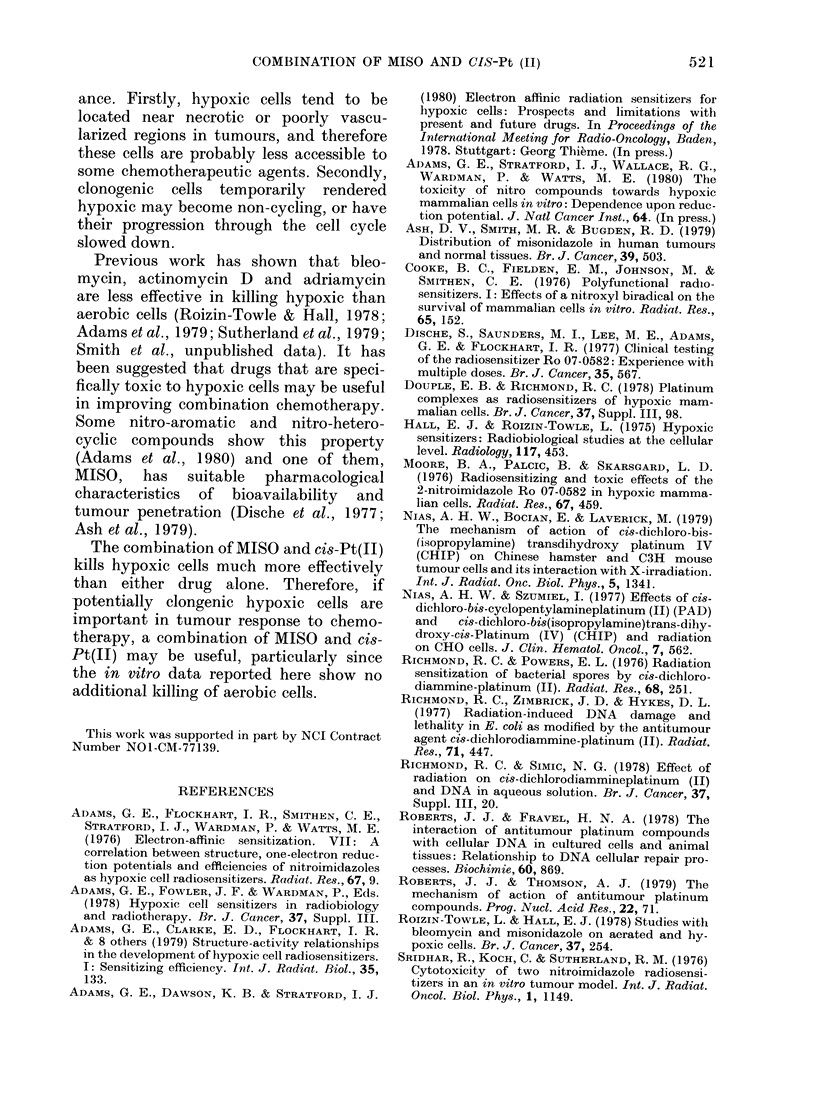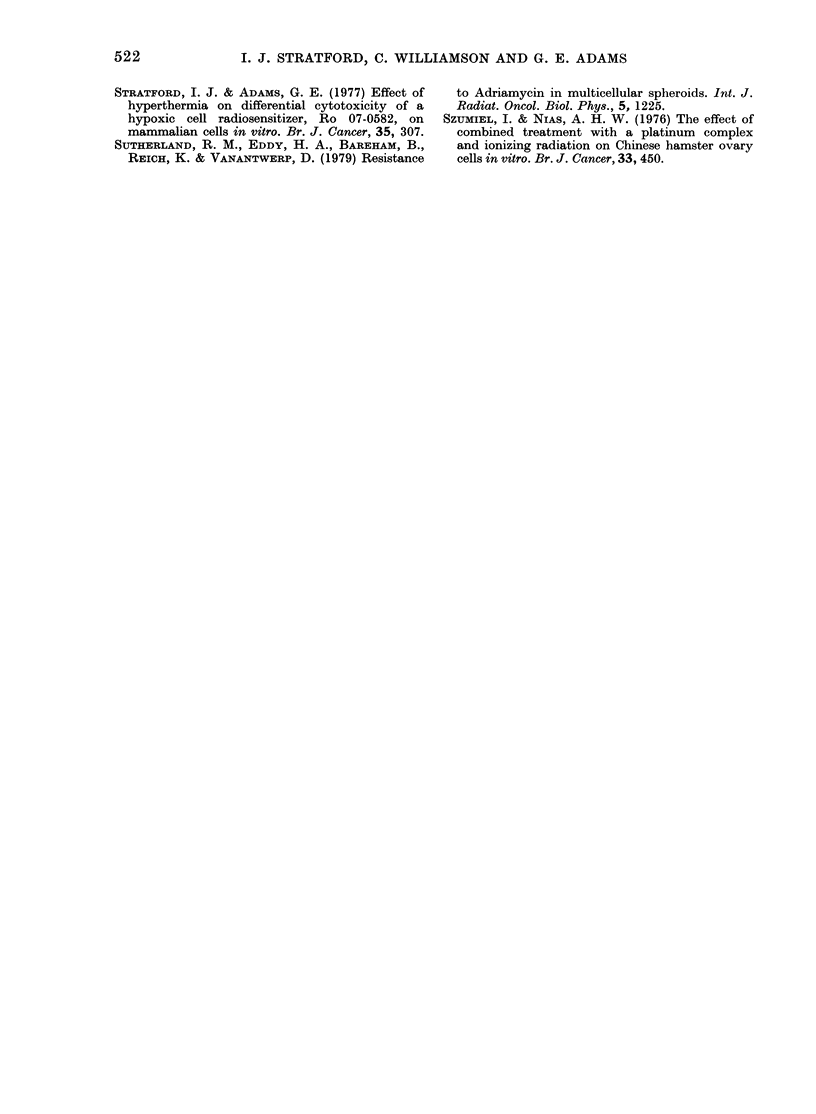# Combination studies with misonidazole and a cis-platinum complex: cytotoxicity and radiosensitization in vitro.

**DOI:** 10.1038/bjc.1980.93

**Published:** 1980-04

**Authors:** I. J. Stratford, C. Williamson, G. E. Adams

## Abstract

cis-Dichlorodiammineplatinum(II) (cis-Pt(II) can act as a radiosensitizer of hypoxic mammalian cells in vitro. Used in combination with misonidazole the level of sensitization achieved is greater than that seen with either drug alone, and it is suggested that these compounds sensitize by independent mechanisms. For cells held at 37 degrees C, cis-Pt(II) shows much greater toxicity to hypoxic cells than to aerobic cells. In combination with misonidazole, no additional cytotoxic effect is shown towards aerobic cells than that seen for cis-Pt(II) alone. However, there is additional killing of hypoxic cells when they are treated with both drugs.


*Br. J. Cancer* (1980) **41**, 517

### COMBINATION STUDIES WITH MISONIDAZOLE AND A *CIS*-PLATINUM COMPLEX: CYTOTOXICITY AND RADIOSENSITIZATION *IN VITRO*

I. J. STRATFORD*, C. WILLIAMSON AND G. E. ADAMS

*From the Radiobiology Unit, Physics Department, Institute of Cancer Research, Sutton, Surrey SM2 5PX, England*

Received 8 November 1979 Accepted 17 December 1979

**Summary.—*cis*-Dichlorodiammineplatinum(II) (*cis*-Pt(II)) can act as a radiosensitizer of hypoxic mammalian cells *in vitro*. Used in combination with misonidazole the level of sensitization achieved is greater than that seen with either drug alone, and it is suggested that these compounds sensitize by independent mechanisms.**

**For cells held at 37°C, *cis*-Pt(II) shows much greater toxicity to hypoxic cells than to aerobic cells. In combination with misonidazole, no additional cytotoxic effect is shown towards aerobic cells than that seen for *cis*-Pt(II) alone. However, there is additional killing of hypoxic cells when they are treated with both drugs.**

THE USE of misonidazole (MISO) as a radiosensitizer of hypoxic cells in tumours is well established (for review see Adams *et al.*, 1978). In addition, a potential role for its use in chemotherapy is being considered because of the ability of this drug to be preferentially cytotoxic towards hypoxic cells (Hall & Roizin-Towle, 1975; Moore *et al.*, 1976; Sridhar *et al.*, 1976; Stratford & Adams, 1977). If MISO is to be of value in chemotherapy it is necessary to use it in combination with drug(s) which can sterilize the aerobic fraction of tumour cells. We are currently studying the cytotoxic effect of some common antineoplastic drugs, including a *cis*-Platinum complex, *cis*-dichlorodiammineplatinum-(II) (*cis*-Pt(II)). *cis*-Pt(II) is a proven cytotoxic agent (see Roberts & Thomson, 1979, for review) and can also act as a radiosensitizer of hypoxic cells (Richmond & Powers, 1976; Richmond *et al.*, 1977; Douple & Richmond, 1978; Nias *et al.*, 1979). Therefore we have examined the radiosensitizing and cytotoxic properties of MISO and *cis*-Pt(II) in combination.

### MATERIALS AND METHODS

*Cells.*—Chinese hamster V79-379A cells used in this work were maintained in spinner culture in Eagles' Minimal Essential Medium (MEM) modified for suspension cultures (Flow Laboratories Ltd.) supplemented with 7·5% foetal calf serum (FCS, Gibco-Biocult Ltd.). Cells were kept in log phase at concentrations ranging between $10^5$ and $10^6$/ml.

*Cytotoxicity experiments.*—250ml spinner flasks were fitted with a gas inlet/outlet system and a sidearm through which samples could be withdrawn. Asynchronous, log-phase cells at a concentration of $2 \times 10^5$/ml were suspended in MEM + 7·5% FCS and held in a water bath at 37°C. The compounds were dissolved in MEM + 7·5% FCS and added to the suspension which was buffered with bicarbonate to pH 7·4. When appropriate, the spinner containing cells was deaerated by flowing $N_2$ plus 5% $CO_2$ (<10 pts/$10^6$ $O_2$; BOC Ltd) at 500 ml/min over the surface of the stirred suspension throughout the experiment. Samples of cells were withdrawn at appropriate times, centrifuged, resuspended, counted, diluted, plated in MEM + 15% FCS and incubated for 7–10 days at 37°C before scoring for colony formation. Further details

* To whom correspondence should be addressed.

of the technique are described elsewhere (Stratford & Adams, 1977).

*Radiation experiments.*—Cells were harvested from log-phase cultures, diluted appropriately and added to glass Petri dishes containing 2·5 ml MEM, supplemented with 15% FCS. Cells were allowed to attach at 37°C for 2–3 h before the medium was removed and replaced with fresh medium containing drug(s). Irradiations with $^{60}Co$ $\gamma$-rays were carried out in "dural" containers which can hold 4 Petri dishes (Cooke *et al.*, 1976). These vessels were made hypoxic by purging with $N_2+5\%$ $CO_2$ (BOC Ltd) for 1 h, after which the vessels were sealed and irradiated at room temperature at a dose rate of 4·2 gray/min. After irradiation, the medium was removed and replaced with fresh MEM + 15% FCS and the cells incubated for 7–9 days before scoring for colony formation.

*Compounds.*—Misonidazole was supplied by Dr C. Smithen of Roche Products Ltd, Welwyn Garden City, Herts, and *cis*-Pt(II) by Dr K. Harrap, Institute of Cancer Research, Sutton, England.

FIG. 1.—Toxicity of 5mM MISO to V79-379A cells *in vitro*. ●, 5mM MISO under hypoxic conditions; ○, 5mM MISO in air; X, cells under hypoxia alone. Data points are means from 7 replicate experiments.

### RESULTS

### *Cytotoxicity studies*

The effect of 5mM MISO (1 mg/ml) on Chinese hamster V79-379A cells held under aerobic or hypoxic conditions is shown in Fig. 1. At this concentration MISO has no cytotoxic effect on cells in air over the duration of the experiments (5 h). In contrast, MISO under hypoxic conditions reduces the surviving fraction to $5\times10^{-3}$. In the absence of MISO, hypoxic cells show no loss of viability.

A similar series of experiments were done with 10μM *cis*-Pt(II) (3 μg/ml) under aerobic and hypoxic conditions at 37°C. These results are shown in Fig. 2. When cells are held in air 10μM *cis*-Pt(II) reduces survival in an exponential manner and after 5 h the surviving fraction of cells is $10^{-2}$. However, under hypoxic conditions cells are very much more sensitive to treatment with *cis*-Pt(II), and after 5 h exposure survival is reduced to $10^{-5}$.

The results of cytotoxicity experiments when 5mM MISO and 10μM *cis*-Pt(II) are used in combination are illustrated in

FIG. 2.—Toxicity of 10μM *cis*-Pt(II) to V79-379A cells *in vitro*. ○, cells under hypoxic conditions (4 replicate experiments); ●, cells in air (2 replicate experiments).

FIG. 3.—Toxicity of the drug combination 10μM *cis*-Pt(II) + 5mM MISO to V79-379A cells *in vitro*. ○, cells under hypoxic conditions (4 replicate experiments); ●, cells in air (2 replicate experiments). The dashed lines are the responses for cells treated with 10μM *cis*-Pt(II) (Fig. 2). The dotted line is the response for hypoxic cells treated with 5mM MISO (Fig. 1).

Fig. 3. In air, the drug combination shows no greater toxicity than *cis*-Pt(II) alone, but under hypoxic conditions the mixture of MISO and *cis*-Pt(II) is considerably more toxic than either drug alone. Simple addition of the cytotoxic effects of each drug indicates that the effect of the drug combination is, at the very least, additive.

*Sensitization experiments*

Following the protocol of previous studies (Adams *et al.*, 1976, 1979) we determined enhancement ratios for radiosensitization of hypoxic Chinese hamster cells *in vitro* by MISO and *cis*-Pt(II). At the concentrations tested, both drugs were non-toxic to non-irradiated cells over a 2h

FIG. 4.—Survival curves for V79-379A cells irradiated under hypoxic conditions. +, hypoxia alone; ○, 5μM *cis*-Pt(II); △, 1mM MISO; ●, 5μM *cis*-Pt(II) + 0·2mM MISO.

FIG. 5.—Dependence of enhancement ratio for irradiated hypoxic V79-379A cells on MISO concentration. Dashed line, MISO alone (Adams *et al.*, 1976); ○, MISO + 5μM *cis*-Pt(II); the dotted line is the enhancement ratio for 5μM *cis*-Pt(II) alone.

contact time in hypoxia at room temperature. Full survival curves, examples of which are shown in Fig. 4, were obtained for each compound at one or two concentrations in $N_2$. Assuming an unchanged extrapolation number, enhancement ratios for other concentrations were calculated from a single survival point (usually between $2 \times 10^{-2}$ and $10^{-1}$) which was obtained by appropriate choice of radiation dose.

Enhancement ratios (ER) obtained for hypoxic cells irradiated in the presence of MISO alone were similar to those reported previously for this cell line (Adams *et al.*, 1976) and this dependence of ER on MISO concentration is shown by the dashed line in Fig. 5. The maximum concentration of *cis*-Pt(II) tested was 5 μM, and this gave routinely an ER of 1·12 (see Fig. 4). For combination studies, the radiation response of hypoxic cells was determined for 5μM *cis*-Pt(II) with concentrations of MISO varying from 10 μM up to 2 mM. Fig. 4 shows a survival curve for hypoxic cells irradiated in the presence of 0·2mM MISO plus 5μM *cis*-Pt(II). This combination results in a survival curve which is identical to that obtained for 1mM MISO alone. Values of ER for 5μM *cis*-Pt(II) with a range of MISO concentrations are shown as the open circles in Fig. 5. Clearly, the ERs for 5μM *cis*-Pt(II) plus MISO are greater than those for MISO alone. In addition, the response curves (Fig. 5) appear parallel, which suggests that the two compounds show a degree of additivity that cannot be explained if they sensitize wholly by the same mechanism(s).

### DISCUSSION

*cis*-Platinum complexes can increase the radiation sensitivity of cells *in vitro* under both aerobic and hypoxic conditions (Richmond & Powers, 1976; Szumiel & Nias, 1976; Nias & Szumiel, 1977; Richmond *et al.*, 1977; Douple & Richmond, 1978) with the greatest effect apparently on the hypoxic cells (Richmond & Powers, 1976; Richmond *et al.*, 1977; Douple & Richmond, 1978; Nias *et al.*, 1979). In the present work 5μM *cis*-Pt(II) increases the radiation sensitivity of hypoxic mammalian cells, giving an ER of 1·12. The combination of *cis*-Pt(II) and MISO gives at least an additive response for radiation sensitization of hypoxic cells. This can be rationalized if we consider that the ER of 5μM *cis*-Pt(II) (1·12) is equivalent to 20μM MISO. On the basis of simple equivalence, if the two compounds are acting similarly, the combination of 5μM *cis*-Pt(II) and 20μM MISO should give an ER similar to that seen for 40μM MISO, *viz.* 1·20. In fact the observed ER is about 1·40, a value that could only be expected if the compounds are operating on different lesions and/or by different mechanism(s). If this effect were to hold for other *cis*-Platinum complexes, and if such complexes in combination with MISO produced no increased toxicity in man, this type of drug combination could substantially increase ERs obtainable in radiotherapy.

We have demonstrated that, as a cytotoxic agent, *cis*-Pt(II) is considerably more toxic to hypoxic cells than to aerobic cells. The difference in toxicity is seen after only 2h exposure to the drug, which is likely to preclude the effect being due to any cell-cycle redistribution induced by hypoxia (Roberts & Fraval, 1978). It is possible that the anaerobic environment allows biochemical reduction of *cis*-Pt(II) to a platinum (I) intermediate. This is a highly reactive species (Richmond & Simic, 1978) which could interact with vital cellular macromolecules. Alternatively, hypoxia may inhibit repair of DNA damage caused by *cis*-Pt(II), and this may also modify radiation sensitivity.

The differential cytotoxicity of *cis*-Pt(II) may be important in influencing the efficacy of the *cis*-platinum complexes used clinically, since there is evidence that hypoxic cells may be resistant to some chemotherapeutic drugs. There are two possible reasons for this hypoxic resistance. Firstly, hypoxic cells tend to be located near necrotic or poorly vascularized regions in tumours, and therefore these cells are probably less accessible to some chemotherapeutic agents. Secondly, clonogenic cells temporarily rendered hypoxic may become non-cycling, or have their progression through the cell cycle slowed down.

Previous work has shown that bleomycin, actinomycin D and adriamycin are less effective in killing hypoxic than aerobic cells (Roizin-Towle & Hall, 1978; Adams *et al.*, 1979; Sutherland *et al.*, 1979; Smith *et al.*, unpublished data). It has been suggested that drugs that are specifically toxic to hypoxic cells may be useful in improving combination chemotherapy. Some nitro-aromatic and nitro-heterocyclic compounds show this property (Adams *et al.*, 1980) and one of them, MISO, has suitable pharmacological characteristics of bioavailability and tumour penetration (Dische *et al.*, 1977; Ash *et al.*, 1979).

The combination of MISO and *cis*-Pt(II) kills hypoxic cells much more effectively than either drug alone. Therefore, if potentially clongenic hypoxic cells are important in tumour response to chemotherapy, a combination of MISO and *cis*-Pt(II) may be useful, particularly since the *in vitro* data reported here show no additional killing of aerobic cells.

This work was supported in part by NCI Contract Number NO1-CM-77139.

REFERENCES

ADAMS, G. E., FLOCKHART, I. R., SMITHEN, C. E., STRATFORD, I. J., WARDMAN, P. & WATTS, M. E. (1976) Electron-affinic sensitization. VII: A correlation between structure, one-electron reduction potentials and efficiencies of nitroimidazoles as hypoxic cell radiosensitizers. *Radiat. Res.*, **67**, 9.

ADAMS, G. E., FOWLER, J. F. & WARDMAN, P., Eds. (1978) Hypoxic cell sensitizers in radiobiology and radiotherapy. *Br. J. Cancer*, **37**, Suppl. III.

ADAMS, G. E., CLARKE, E. D., FLOCKHART, I. R. & 8 others (1979) Structure-activity relationships in the development of hypoxic cell radiosensitizers. I: Sensitizing efficiency. *Int. J. Radiat. Biol.*, **35**, 133.

ADAMS, G. E., DAWSON, K. B. & STRATFORD, I. J. (1980) Electron affinic radiation sensitizers for hypoxic cells: Prospects and limitations with present and future drugs. In *Proceedings of the International Meeting for Radio-Oncology, Baden*, 1978. Stuttgart: Georg Thième. (In press.)

ADAMS, G. E., STRATFORD, I. J., WALLACE, R. G., WARDMAN, P. & WATTS, M. E. (1980) The toxicity of nitro compounds towards hypoxic mammalian cells *in vitro*: Dependence upon reduction potential. *J. Natl Cancer Inst.*, **64**. (In press.)

ASH, D. V., SMITH, M. R. & BUGDEN, R. D. (1979) Distribution of misonidazole in human tumours and normal tissues. *Br. J. Cancer*, **39**, 503.

COOKE, B. C., FIELDEN, E. M., JOHNSON, M. & SMITHEN, C. E. (1976) Polyfunctional radiosensitizers. I: Effects of a nitroxyl biradical on the survival of mammalian cells *in vitro*. *Radiat. Res.*, **65**, 152.

DISCHE, S., SAUNDERS, M. I., LEE, M. E., ADAMS, G. E. & FLOCKHART, I. R. (1977) Clinical testing of the radiosensitizer Ro 07-0582: Experience with multiple doses. *Br. J. Cancer*, **35**, 567.

DOUPLE, E. B. & RICHMOND, R. C. (1978) Platinum complexes as radiosensitizers of hypoxic mammalian cells. *Br. J. Cancer*, **37**, Suppl. III, 98.

HALL, E. J. & ROIZIN-TOWLE, L. (1975) Hypoxic sensitizers: Radiobiological studies at the cellular level. *Radiology*, **117**, 453.

MOORE, B. A., PALCIC, B. & SKARSGARD, L. D. (1976) Radiosensitizing and toxic effects of the 2-nitroimidazole Ro 07-0582 in hypoxic mammalian cells. *Radiat. Res.*, **67**, 459.

NIAS, A. H. W., BOCIAN, E. & LAVERICK, M. (1979) The mechanism of action of *cis*-dichloro-bis-(isopropylamine) transdihydroxy platinum IV (CHIP) on Chinese hamster and C3H mouse tumour cells and its interaction with X-irradiation. *Int. J. Radiat. Onc. Biol. Phys.*, **5**, 1341.

NIAS, A. H. W. & SZUMIEL, I. (1977) Effects of *cis*-dichloro-*bis*-cyclopentylamineplatinum (II) (PAD) and *cis*-dichloro-*bis*(isopropylamine)trans-dihydroxy-*cis*-Platinum (IV) (CHIP) and radiation on CHO cells. *J. Clin. Hematol. Oncol.*, **7**, 562.

RICHMOND, R. C. & POWERS, E. L. (1976) Radiation sensitization of bacterial spores by *cis*-dichlorodiammine-platinum (II). *Radiat. Res.*, **68**, 251.

RICHMOND, R. C., ZIMBRICK, J. D. & HYKES, D. L. (1977) Radiation-induced DNA damage and lethality in *E. coli* as modified by the antitumour agent *cis*-dichlorodiammine-platinum (II). *Radiat. Res.*, **71**, 447.

RICHMOND, R. C. & SIMIC, N. G. (1978) Effect of radiation on *cis*-dichlorodiammineplatinum (II) and DNA in aqueous solution. *Br. J. Cancer*, **37**, Suppl. III, 20.

ROBERTS, J. J. & FRAVEL, H. N. A. (1978) The interaction of antitumour platinum compounds with cellular DNA in cultured cells and animal tissues: Relationship to DNA cellular repair processes. *Biochimie*, **60**, 869.

ROBERTS, J. J. & THOMSON, A. J. (1979) The mechanism of action of antitumour platinum compounds. *Prog. Nucl. Acid Res.*, **22**, 71.

ROIZIN-TOWLE, L. & HALL, E. J. (1978) Studies with bleomycin and misonidazole on aerated and hypoxic cells. *Br. J. Cancer*, **37**, 254.

SRIDHAR, R., KOCH, C. & SUTHERLAND, R. M. (1976) Cytotoxicity of two nitroimidazole radiosensitizers in an *in vitro* tumour model. *Int. J. Radiat. Oncol. Biol. Phys.*, **1**, 1149.

STRATFORD, I. J. & ADAMS, G. E. (1977) Effect of hyperthermia on differential cytotoxicity of a hypoxic cell radiosensitizer, Ro 07-0582, on mammalian cells *in vitro*. *Br. J. Cancer*, **35**, 307.

SUTHERLAND, R. M., EDDY, H. A., BAREHAM, B., REICH, K. & VANANTWERP, D. (1979) Resistance to Adriamycin in multicellular spheroids. *Int. J. Radiat. Oncol. Biol. Phys.*, **5**, 1225.

SZUMIEL, I. & NIAS, A. H. W. (1976) The effect of combined treatment with a platinum complex and ionizing radiation on Chinese hamster ovary cells *in vitro*. *Br. J. Cancer*, **33**, 450.